# The Silent Killer: An Autopsy Case of Basilar Artery Thrombosis With Diabetes Mellitus, Metabolic Dysfunction-Associated Steatotic Liver Disease, and Gastroesophageal Reflux Disease

**DOI:** 10.7759/cureus.83701

**Published:** 2025-05-08

**Authors:** Rina Izumi, Koji Hayashi, Ei Kawahara, Asuka Suzuki, Yuka Nakaya, Maho Hayashi, Mamiko Sato, Yasutaka Kobayashi

**Affiliations:** 1 Department of Rehabilitation Medicine, Fukui General Hospital, Fukui, JPN; 2 Department of Pathology, Fukui General Hospital, Fukui, JPN; 3 Department of Internal Medicine, Fukui General Hospital, Fukui, JPN; 4 Graduate School of Health Science, Fukui Health Science University, Fukui, JPN

**Keywords:** basilar artery thrombosis, cerebral thrombosis, “diabetes mellitus”, gastroesophageal reflux disease (gerd), metabolic dysfunction-associated steatotic liver disease (masld), non-alcoholic steatohepatitis (nash)

## Abstract

We report a case of autopsy-confirmed basilar artery thrombosis in a 58-year-old male with a history of diabetes, metabolic dysfunction-associated steatotic liver disease (MASLD), and gastroesophageal reflux disease (GERD). The patient presented with cardiopulmonary arrest while eating and died two hours after the initial collapse. Postmortem imaging could not identify a definitive cause of death. The autopsy revealed acute hemorrhagic infarcts in the brainstem, along with evidence of basilar artery thrombosis and severe arteriosclerosis. The presence of diabetes, MASLD, and GERD may have contributed to the development of arteriosclerosis and increased the risk of cardiovascular events. This case highlights the complex interplay between seemingly unrelated medical conditions and their potential to contribute to sudden and unexpected death. While diabetes and MASLD are often referred to as "silent killers," in this case, basilar artery thrombosis acted as the final "silent killer" for the patient. The autopsy revealed a complex web of silent killers, ultimately concluding that basilar artery embolism was the cause of death.

## Introduction

Basilar artery thrombosis, leading to basilar artery occlusion (BAO), is a significant complication in stroke cases. Although the exact incidence of BAO is not precisely known, it is estimated that this condition accounts for approximately 1% of all stroke occurrences [1]. Patients experiencing BAO may exhibit a range of symptoms, from mild issues like nausea and dizziness to more severe complications such as aphasia, dysphagia, loss of consciousness, or even life-threatening states if not treated promptly [2,3]. Therefore, obtaining imaging studies that include computed tomography (CT), computed tomography angiography (CTA), and diffusion-weighted magnetic resonance imaging (MRI) as part of the diagnostic approach is crucial to make an accurate diagnosis [4]. In particular, MRI has higher sensitivity because it is challenging for CT to detect acute cerebral infarction lesions, including BAO, during the acute phase. Rapid intervention, including the use of intra-arterial thrombolytics and mechanical thrombectomy when appropriate, has been shown to enhance clinical outcomes in eligible patients [5].

This condition arises from the blockage of blood flow in the posterior circulation, primarily due to atherosclerosis or thromboembolic events [6]. Mechanisms of ischemic stroke, such as atherosclerosis and cardioembolism stemming from various heart conditions, contribute to BAO by disrupting blood flow and causing infarctions in areas supplied by the basilar artery [7]. Atherosclerosis is driven by factors including dyslipoproteinemia and hypertension, leading to injury of the vessel wall that can promote thrombus formation [8]. Additionally, conditions like COVID-19, specific vascular malformations, and hematological disorders have been linked to an elevated risk of BAO [8].

We report a case of autopsy-confirmed basilar artery thrombosis with diabetes, metabolic dysfunction-associated steatotic liver disease (MASLD), and gastroesophageal reflux disease in a patient who presented to our hospital with cardiopulmonary arrest (CPA).

## Case presentation

A 58-year-old male, with a history of diabetes and psychiatric disorders, developed a cardiopulmonary arrest while having breakfast at home. Choking on food was suspected, and his family immediately called emergency services. During transport in the ambulance, chest compressions and endotracheal intubation were performed. Despite receiving three doses of adrenaline, a return of spontaneous circulation (ROSC) was not achieved.

Upon arrival at the hospital, 40 minutes after the collapse, both pupils were dilated (5/5 mm) and non-reactive to light bilaterally. The doll's eye reflex was negative, with the eyes moving in the same direction even when the head was moved rapidly up, down, left, or right. After administering an additional dose of adrenaline, ROSC was finally achieved. A bronchoscopy was performed to remove an airway obstruction caused by a foreign body. However, despite noting some residue in the mouth, no foreign body was found in the airway. Although ROSC was restored, spontaneous respiration did not resume, and the family declined mechanical ventilation. Bag-valve-mask (BVM) ventilation was discontinued, and oxygen therapy via a face mask was initiated for observation. Shortly afterward, cardiac arrest recurred, and death was confirmed two hours after the initial collapse.

**Figure 1:**
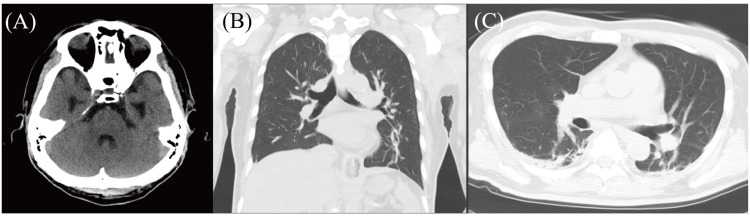
Postmortem computed tomography (CT) imaging (A) No brainstem lesions or fresh cerebral infarctions were detected in any areas of the brain. (B, C) No food or other foreign body was noted in the main bronchus or trachea.

Autopsy brain imaging with computed tomography (CT) revealed no significant findings, and neither brainstem lesions nor fresh cerebral infarctions were detected in any areas of the brain (Figure 1A). No food or other foreign body was noted in the main bronchus or trachea (Figures 1B-1C).

Pathological findings

**Figure 2:**
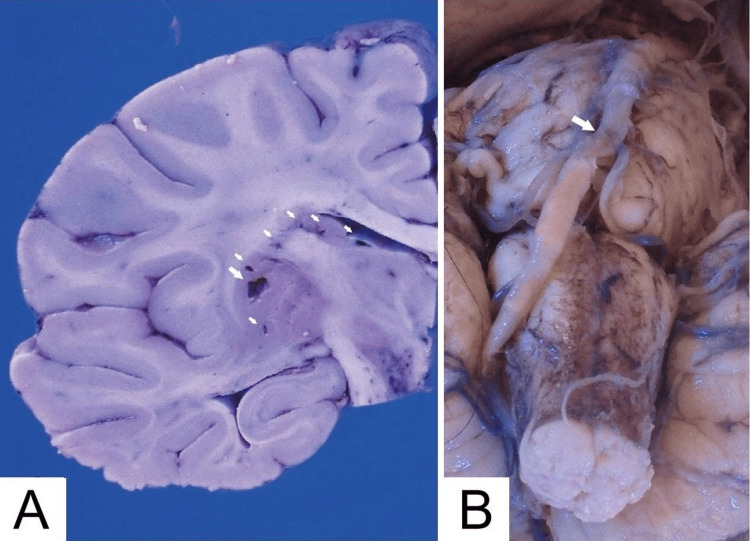
Gross examination of the brain (A) Coronal section of the cerebrum through the basal ganglia. Multiple lacunar infarcts (arrows) are present in the putamen, internal capsule, and head of the caudate nucleus. (B) Ventral view of the pons and medulla oblongata. The basilar artery and right vertebral artery are calcified, contrasting with the hypoplastic left vertebral artery. Intraluminal thrombus (arrow) is evident in the non-calcified regions near the junction of the left and right vertebral arteries with the basilar artery.

The brain weighed 1230 g. Gross examination revealed multiple lacunar infarctions in the putamen, internal capsule, caudate nucleus, pons, deep white matter, and occipital cortex (Figure 2A). The walls of the basilar artery and right vertebral artery were calcified (Figure 2B). The liver, spleen, and kidneys exhibited congestion. Atherosclerotic changes were also noted in the anterior cerebral artery, middle cerebral artery, aorta, and coronary arteries.

**Figure 3:**
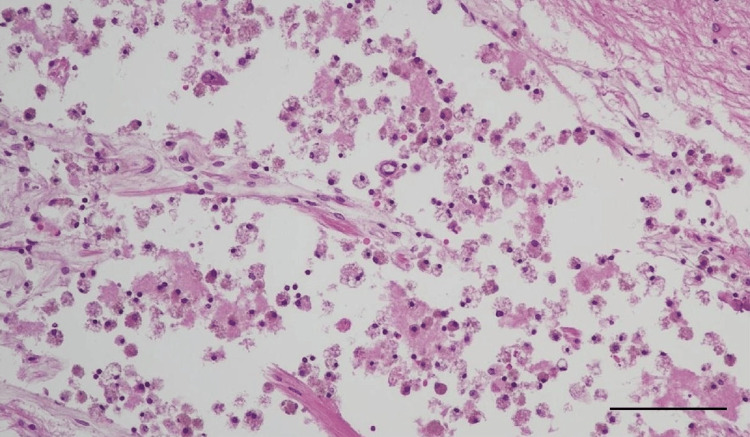
Lacunar infarct in the basal ganglia Accumulation of foamy macrophages within a lacunar infarct. H&E stain. Scale bar: 100 µm.

**Figure 4:**
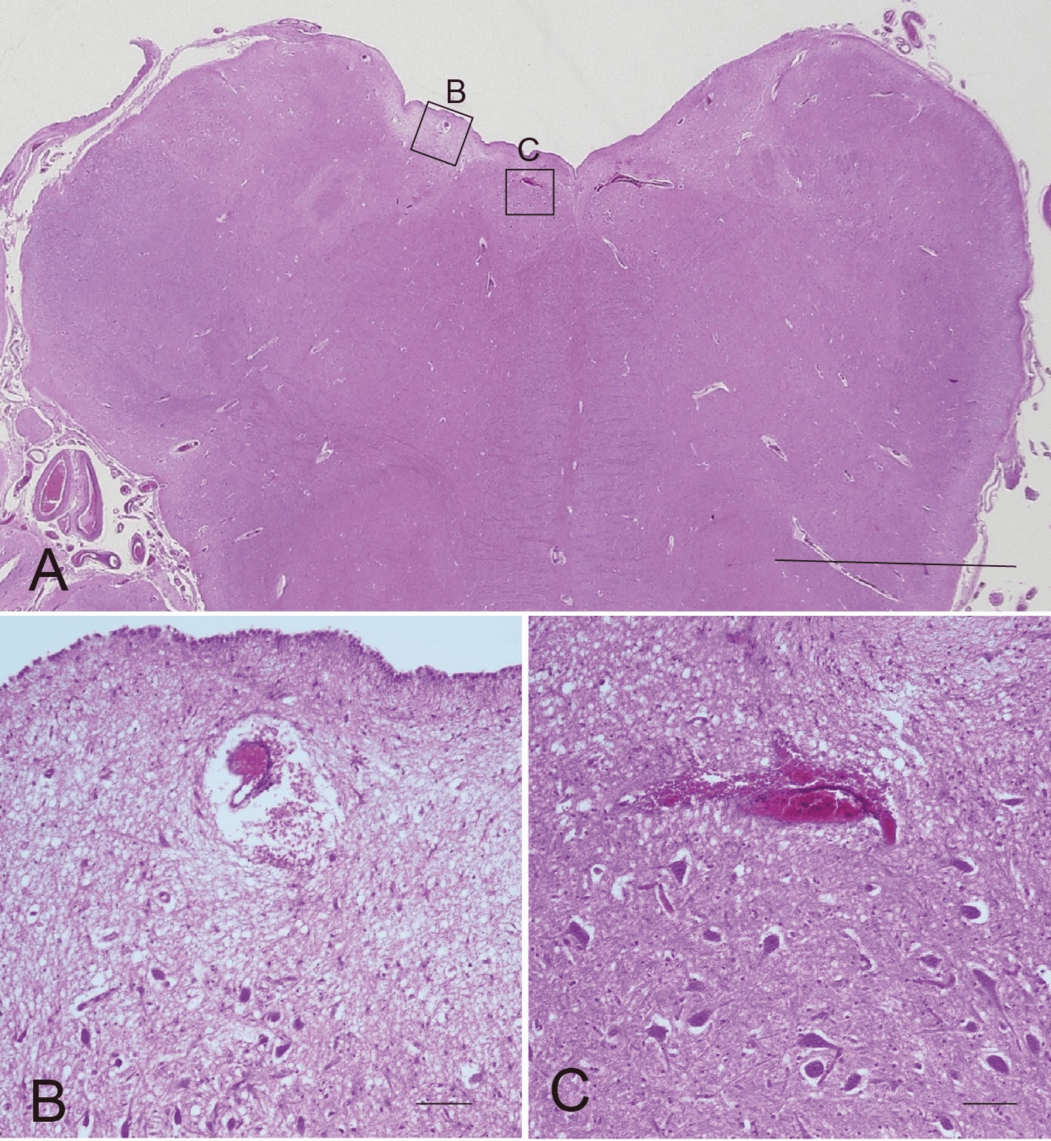
Fresh minute hemorrhagic infarcts in the medulla oblongata (A) Low magnification of the medulla oblongata. Left and right (square B) nuclei of hypoglossal nerves and the right dorsal nucleus of the vagus nerve (square C) were detected. H&E stain. Scale bar: 1 cm. (B) Higher magnification of hemorrhagic microinfarct with microthrombus in the nucleus of the vagus nerve. Scale bar: 50 µm. (C) Higher magnification of hemorrhagic microinfarct with microthrombus in the nucleus of the hypoglossal nerve. H&E stain. Scale bar: 50 µm.

Microscopic examination demonstrated old anemic infarcts containing foamy macrophages within the lumen, consistent with multiple lacunar infarctions (Figure 3). Additionally, acute hemorrhagic infarcts, histologically characterized by intracapillary thrombi with perivascular hemorrhage, were detected in the bilateral nuclei of hypoglossal nerves, right dorsal nucleus of the vagus nerve, and right intramedullary vagus nerve (Figures 4A-4C).

**Figure 5:**
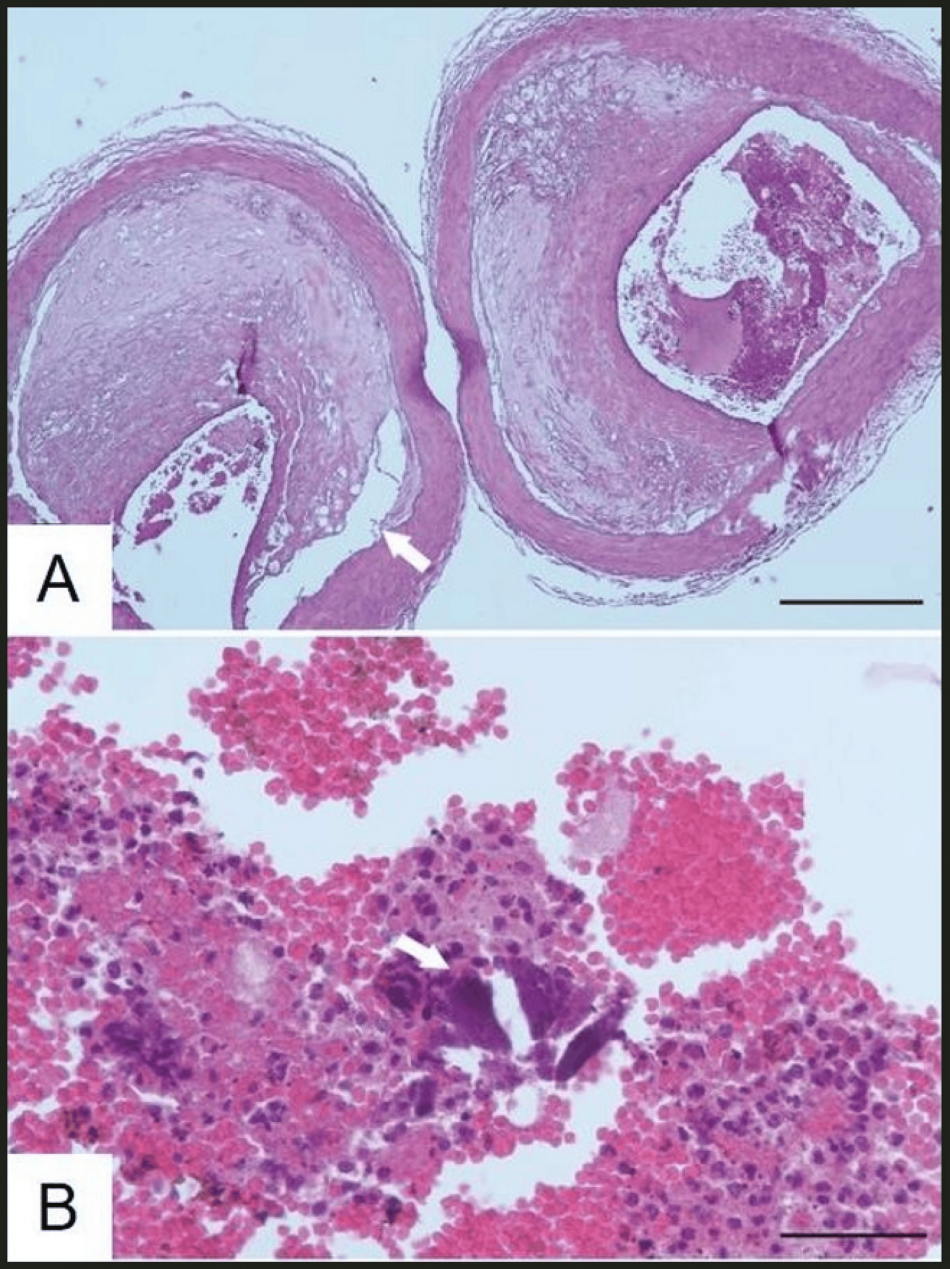
Thrombosis and severe atherosclerosis of the basilar and right vertebral arteries (A) Transverse sections of the lowermost portion of the basilar artery and the uppermost portion of the right vertebral artery. Thrombus and eccentric stenosis due to atherosclerotic plaque are present. The atheromatous plaque is ruptured (arrow) in the left vertebral artery. Scale bar: 500 µm. (B) Higher magnification of the thrombus. Numerous neutrophils are infiltrated into the thrombus, suggesting it formed at least one day prior. A minute calcified fragment is also present (arrow). Scale bar: 100 µm.

Reexamination of the basilar artery and vertebral artery revealed severe arteriosclerosis and eccentric stenosis with atherosclerotic plaque and thrombus (Figure 5A). Microscopic examination confirmed eccentric stenosis with atherosclerotic plaque and thrombus. The plaque was ruptured, and minute calcific fragments were involved in the intraluminal thrombus (Figure 5B). The thrombus contained numerous neutrophils, indicating it had formed at least one day prior. Immunostaining for α-synuclein did not reveal any Lewy bodies.

**Figure 6:**
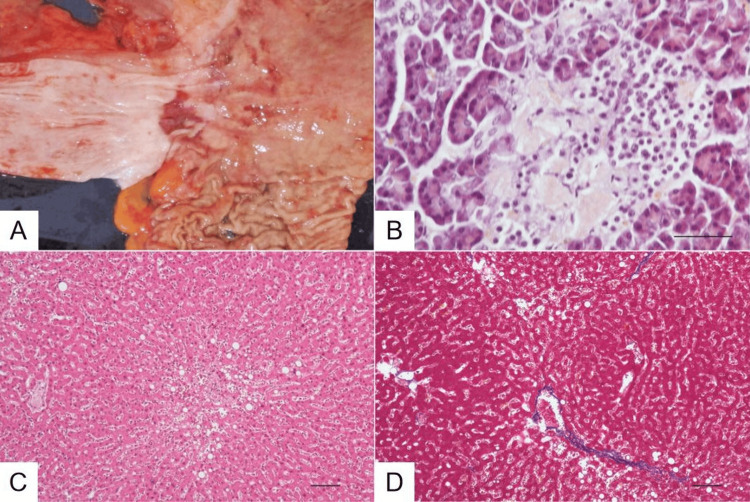
Macroscopic and microscopic findings (A) Esophagogastric junction. Geographical erosion at the lower esophagus. Scale bar: 1 cm. (B) Langerhans islet. Congo red-positive amyloid is deposited in the Langerhans islet. Congo red stain. Scale bar: 50 µm. (C) Liver showing steatosis. H&E stain. Scale bar: 100 µm. (D) Liver showing bridging fibrosis. Azan stain. Scale bar: 100 µm.

Additionally, the esophagus showed an erosion near the esophagogastric junction (Figure 6A), attributed to reflux esophagitis. Amyloid deposition was observed in the pancreatic islets of Langerhans (Figure 6B). MASLD was identified (Figures 6C, 6D).

Based on the autopsy findings, the direct cause of death was determined to be basilar artery thrombosis, which led to medullary infarction and subsequently resulted in respiratory and circulatory dysfunction.

## Discussion

We present a case of CPA followed by an autopsy-confirmed diagnosis of basilar artery thrombosis. The patient had a history of major tranquilizer use, gait disturbance, and choking, leading emergency physicians to suspect medication-induced Parkinsonism and potential suffocation as causes of the CPA. However, autopsy imaging with CT revealed no foreign objects in the airways, including the trachea. Additionally, brain autopsy imaging was unremarkable, with no evidence of acute ischemia. This unexpected finding prompted a complete autopsy, including the brain, which ultimately revealed the underlying cause of the killer: basilar artery thrombosis.

Here, we summarize the systemic and brain pathological findings. In the systemic examination, autopsy revealed amyloid deposits in the pancreas, supporting the diagnosis of type 2 diabetes mellitus [9]. Additionally, liver tissue exhibited fatty infiltration and mononuclear cell infiltration, indicating MASLD, previously referred to as nonalcoholic steatohepatitis (NASH). The aorta showed evidence of atherosclerosis, along with significant atherosclerotic changes in the anterior cerebral artery, middle cerebral artery, and coronary arteries. Brain pathology revealed that the substantia nigra was preserved, and although there was nonspecific staining by α-synuclein immunostaining, no obvious Lewy bodies were identified. Foamy macrophages were observed in areas perfused by the vertebral basilar artery, suggesting acute ischemic stroke. Thrombus formation and severe atherosclerosis were found in the vertebral basilar artery, and acute hemorrhagic infarcts were detected in the bilateral nuclei of the hypoglossal nerves and the right dorsal nucleus of the vagus nerve.

DM is associated with several serious complications, which can be categorized into micro-vascular and macro-vascular complications [10]. Micro-vascular complications include retinopathy, nephropathy, and neuropathy, while macro-vascular complications include ischemic heart disease, stroke, and peripheral vascular disease. Therefore, DM is related to very serious health issues, which is why many experts refer to it as the 'silent killer’ [10].

MASLD is also known as a ‘silent killer’ that can advance to metabolic dysfunction-associated steatohepatitis (MASH), a more serious condition [11]. Obesity is a significant factor in the onset and advancement of MASLD, which is frequently linked to metabolic syndrome and insulin resistance [11]. As a result, MASLD is closely associated with both diabetes and arteriosclerosis. Notably, a previous study revealed that NASH is known to increase the risk of developing ischemic stroke [12].

GERD occurs when stomach contents repeatedly flow back into the esophagus, causing bothersome symptoms and/or complications [13]. Esophageal complications related to GERD include erosive esophagitis, esophageal hemorrhage, ulceration, strictures, and Barrett's esophagus [14]. Notably, previous studies have revealed that GERD increases the risk of coronary atherosclerosis [15]. The mechanism by which GERD may affect the cardiovascular system involves shared neurological control between the esophagus and coronary arteries, where gastroesophageal acid reflux via the vagal reflex can lead to coronary hypoperfusion [13]. This condition may relax the lower esophageal sphincter, exacerbating reflux and increasing the risk of coronary artery disease. Additionally, GERD may increase the risk of ischemic stroke, especially in patients with atrial fibrillation [15]. GERD, particularly esophagitis, may increase the risk of AF through three proposed mechanisms: esophageal inflammation, autonomic nerve activation, and mechanical irritation that affects the nearby left atrium [15]. While proton pump inhibitors (PPIs) are used for the treatment of GERD, their effects on cardiovascular diseases are controversial. PPIs may reduce cardiac contractility and elevate homocysteine levels, further raising atherosclerosis risk [13]. However, PPIs have been shown to alleviate AF symptoms, suggesting an indirect relationship between GERD and AF [15].

In our case, post-mortem brain pathology revealed basilar artery thrombosis and fresh cerebral infarcts in its perfusion area, confirming the cause of death. Additionally, the postmortem pathological examination revealed DM, MASLD, and GERD, conditions associated with arteriosclerosis and/or arrhythmia. Based on the pathological findings, the patient experienced a basilar artery embolism during breakfast, leading to cardiopulmonary arrest. The patient experienced sudden death due to the "silent killer" of basilar artery thrombosis, believed to have occurred as a result of indirect mechanisms related to other "silent killers," including DM, MASLD, and GERD. Further studies are needed to elucidate the underlying relationship between basilar artery thrombosis, DM, MASLD, and GERD.

This report has some limitations. The patient was initially admitted to our hospital via ambulance, and a complete medical history was not available. Autopsy revealed previously undiagnosed conditions, including diabetes, MASLD, and arteriosclerosis, but the patient's medical records regarding these conditions and his history of GERD and hypertension were not accessible. Additionally, although the patient had a history of psychiatric disorder, we did not have access to those records. The lack of complete medical information limits the detailed description of the patient's history. Furthermore, brain pathology revealed a previous cerebral infarction lesion, but a clear history of transient ischemic attack or stroke remains unclear. With access to a comprehensive medical history, our presentation would have been more informative. In addition, no foreign body was found in the airway during bronchoscopy after the patient arrived at our hospital; however, the ambulance team had already performed emergency interventions, including endotracheal intubation, prior to the patient's arrival. Given that infarction of the vagus and hypoglossal nuclei due to BAO was noted, suffocation may have been an indirect contributing factor to the cause of death.

## Conclusions

This case highlights the complex interplay between seemingly unrelated medical conditions and their potential for contributing to sudden, unexpected death. While the immediate cause of death was confirmed to be BAO due to basilar artery thrombosis, postmortem findings revealed a constellation of conditions that likely played a significant role in this tragic outcome. The presence of DM, MASLD, and GERD suggests a potential link to the development of arteriosclerosis and/or arrhythmias. These medical conditions may act as 'silent killers' that increase the risk of cardiovascular events, such as stroke, which ultimately contributed to the patient’s death.

While the exact mechanisms by which these conditions contributed to the BAO remain unclear, this case underscores the importance of comprehensive medical evaluations, particularly in individuals with risk factors for cardiovascular disease. Furthermore, ongoing vascular follow-up and management of these silent killers are essential in slowing down the progression of atherosclerosis, thereby potentially preventing similar outcomes in the future.
